# Teacher-student perceptual mismatch in positive co-experiences is associated with adolescent depression, bullying victimization, and suicidality

**DOI:** 10.3389/fpubh.2026.1976771

**Published:** 2026-09-30

**Authors:** Yuanyuan Wang, Hongyu Shi, Xiang Gu, Zhihong Tan, Junqi Ma, Ke Wang

**Affiliations:** 1 Department of Pediatrics, Beijing Haidian Hospital, Haidian Section of Peking University Third Hospital, Beijing, China; 2 Department of Microbiology and Biochemical Pharmacy, Shandong University of Commerce and Technology, Jinan, Shandong, China; 3 Department of Medical Affairs, Heze Municipal Hospital, Heze, Shandong, China; 4 Department of Psychiatry, Heze Municipal Hospital, Heze, Shandong, China

**Keywords:** adolescent mental health, bullying victimization, multi-informant assessment, perceptual mismatch, positive co-experiences, self-determination theory, suicidality, teacher-student relationship

## Abstract

**Background:**

Positive teacher-student interactions may be associated with adolescent mental health, but teachers and students may not experience the same interactions similarly. Integrating self-determination theory with the Operations Triad Model, we examined whether mismatch in positive teacher-student co-experiences was associated with adolescent mental health and whether mismatch direction mattered.

**Methods:**

Cross-sectional paired data were analyzed from 1,836 students aged 11–17 years nested within 40 homeroom teachers in Hunan Province, China. Students and teachers completed parallel three-item measures of positive co-experiences. Four profiles were defined using reporter-specific medians: both high, teacher high-student low, both low, and teacher low-student high. Generalized estimating equations accounted for classroom clustering and adjusted for demographic covariates.

**Results:**

Homeroom-teacher ratings showed little convergence with class-mean student ratings (*r* = −0.186, *P* = 0.250). Compared with the both-high profile, the teacher high-student low profile showed higher depressive symptoms, bullying severity, odds of any bullying victimization (OR = 1.343, 95% CI 1.129–1.598), and odds of suicidality (OR = 1.729, 95% CI 1.183–2.526). Directional mismatch was especially informative: relative to the teacher low-student high profile, the teacher high-student low profile had higher depressive symptoms (beta = 0.665), higher bullying severity (beta = 0.730), 1.79-fold higher odds of bullying victimization, and 2.04-fold higher odds of suicidality. Extreme-discrepancy sensitivity analyses yielded the same pattern.

**Conclusion:**

Teacher-student perceptual mismatch was associated with adolescent mental health in a direction-specific manner. The findings are consistent with the proposition that positive relational experiences may be most protective when teacher-perceived support is also experienced positively by students, and that multi-informant discrepancies can provide useful contextual information for school-based mental health screening, rather than constituting clinical diagnostic measures.

## 1 Introduction

Mental health problems during adolescence are a major public health concern. In China, a recent global burden of disease analysis estimated that approximately 30.8 million children and adolescents were living with mental disorders in 2021, while a large multisite epidemiological study based on detailed clinical assessments reported a weighted prevalence of 17.5% for any mental disorder among youths aged 6–16 years (1, 2). Depression and anxiety occupy central positions in symptom networks of Chinese adolescents (3), and bullying victimization is among the environmental exposures most consistently associated with adolescent self-harm and suicidality (4). These findings underscore the need to identify modifiable relational conditions within everyday developmental settings, particularly schools.

Teacher-student relationships represent one such condition. A 2025 second-order meta-analytic review synthesizing 26 meta-analyses and approximately 2.64 million students found that teacher-student relationship quality was consistently associated with a broad range of student outcomes, including wellbeing, school belonging and engagement, behavior, and academic functioning (5). Complementary systematic reviews indicate that stronger school connectedness is associated with lower subsequent depression and anxiety (6) and with lower suicidal ideation among high school students (7). Yet most research operationalizes relational quality from a single perspective—typically either teacher reports or student reports alone—rather than integrating both informants, even though teachers and students occupy different positions within the same classroom and may not experience the same interactions in the same way. Whether teacher-student perceptual incongruence itself carries meaningful information about adolescent mental health remains understudied.

Self-determination theory (SDT), particularly basic psychological need theory (BPNT), provides a framework for understanding why the student's experience of relational support may matter. SDT proposes that social contexts promote adaptive functioning when they support the needs for autonomy, competence, and relatedness, whereas active need frustration is associated with maladjustment (8, 9). In school settings, teachers can provide need-supportive conditions through autonomy support, competence-affirming feedback, and warm interpersonal involvement. Longitudinal evidence in Chinese adolescents indicates that teacher autonomy support predicts greater psychological-need satisfaction and school engagement, which in turn predict lower anxiety and depression (10). In addition, an SDT-based study of referred and non-referred adolescents found that need frustration was associated with suicidal ideation through anxious-depressive symptoms (11). Together, these findings suggest that potentially supportive teacher behavior may be psychologically consequential only to the extent that it is experienced by students as meaningful support.

Positive teacher-student co-experiences provide a concrete relational context in which these processes may unfold. Ding et al. demonstrated across questionnaire, field-experimental, and laboratory studies that shared learning and problem solving, celebrating student achievements, and making progress through joint effort can strengthen teacher-student relationships through emotional bonding (12). In a recent study from the same Hunan adolescent research program, Zhang et al. incorporated positive teacher-student co-experiences into a multidimensional risk network for adolescent self-injurious behavior alongside depression, bullying victimization, and other psychosocial factors (13). However, whether homeroom teachers and students converge in their perceptions of these positive experiences, and whether non-convergence is clinically informative, remains unclear.

The operations triad model (OTM) of multi-informant assessment offers a complementary framework for this question. The OTM argues that low convergence between informants should not automatically be treated as measurement error; when discrepancies show systematic associations with external criteria, they may represent meaningful ‘diverging operations' (14). Subsequent work has further emphasized the value of preserving and testing informant discrepancies in youth mental-health assessment (15). In school settings, teacher-student incongruence in perceptions of school equity has been associated with lower student connectedness (16), while profile-based parent-adolescent research suggests that the direction of disagreement can also be informative (17). Integrating SDT with the OTM therefore yields a specific proposition: a teacher-high/student-low profile may mark a gap between the relational support that teachers believe is present and the support that students actually experience, and this directional gap may be more relevant to mental health than disagreement *per se*.

Accordingly, the present study used paired reports from Chinese adolescents and their homeroom teachers to examine perceptual profiles of positive teacher-student co-experiences in relation to depressive symptoms, bullying victimization, and suicidality. Rather than treating teacher-student relationship quality as a unidimensional construct, we treated perceptual mismatch itself as a potentially informative risk marker. We distinguished four profiles: both high, teacher high-student low, both low, and teacher low-student high. We tested two *a priori* hypotheses: (H1) the both-high profile would show comparatively favorable mental health relative to the other profiles; and (H2) adolescents in the teacher high-student low profile would show poorer mental health than adolescents in the reverse, teacher low-student high profile (directional mismatch hypothesis).

## 2 Materials and methods

### 2.1 Participants and study design

This study used data from the EPOCH-A cross-sectional survey of adolescents in Hunan Province, China. The original student database included 2,042 students aged 11–17 years from 44 classes. Teacher questionnaires were completed by the homeroom teacher of each class. Student records were matched to the self-report of their own homeroom teacher; therefore, students within the same class shared the same teacher-level rating. Because homeroom teachers rated their overall interactions with the entire class whereas each student rated individual experience, the resulting perceptual mismatch should be understood as a cross-level discrepancy between class-level teacher appraisal and individual student experience, rather than a fully symmetric teacher-student dyadic discrepancy. After excluding three classes without valid teacher linkage and one class with invalid teacher data, 1,859 students nested within 40 classes/homeroom teachers remained. A further 23 students with incomplete positive co-experience data were excluded, leaving 1,836 students for the primary analyses.

The survey was administered at school using standardized procedures by trained research personnel and school staff. The study was approved by the Ethics Committee of the National Clinical Research Center for Mental Disorders, Department of Psychiatry, the Second Xiangya Hospital of Central South University (approval no. (2023) Guo Lun Shen [Ke] No. 036) and was conducted in accordance with the Declaration of Helsinki. Written informed consent was obtained from participating homeroom teachers, students, and students' parents or legal guardians before data collection. Participation was voluntary, and students could decline any question or withdraw at any time.

### 2.2 Measures

#### 2.2.1 Positive teacher-student co-experiences

Positive teacher-student co-experiences were assessed using three items derived from Ding et al. (12). Students and homeroom teachers completed parallel items from their respective perspectives, covering shared learning/problem solving, teachers celebrating students' achievements, and joint teacher-student efforts leading to progress. A sample student item was, 'My teacher learns and solves problems together with us.' Items were rated from 1 (never) to 4 (frequently) and averaged; higher scores indicated more positive co-experiences. Cronbach's alpha was 0.868 for student reports and 0.796 for homeroom-teacher reports.

#### 2.2.2 Teacher-student perceptual profiles

Consistent with profile-based approaches to multi-informant discrepancy (16, 17), teacher and student scores were separately dichotomized at their sample medians to produce an interpretable primary profile analysis. This four-profile approach was selected because it provides a direction-specific classification that directly maps onto the study's core theoretical question—whether the direction of mismatch carries differential information for mental health. We acknowledge that median dichotomization inevitably sacrifices some information and may assign individuals with similar scores to different categories near the cutoff; to address this concern, a prespecified sensitivity analysis using standardized discrepancy scores and extreme quartiles was conducted (see Section 2.4). The median homeroom-teacher score was 3.075 and the median student score was 3.33. Combining the two ratings yielded four profiles: both high (teacher high-student high), teacher high-student low, both low (teacher low-student low), and teacher low-student high. The both-high group served as the primary reference category. A prespecified contrast directly compared the two discordant profiles (teacher high-student low vs. teacher low-student high) to test the *a priori* directional mismatch hypothesis (H2).

#### 2.2.3 Depressive symptoms

Depressive symptoms were assessed with two items corresponding to the core PHQ-2 symptoms of anhedonia and depressed mood during the previous 2 weeks (18). The survey used four frequency categories, which were recoded from 1–4 to 0–3 and summed (range, 0–6); higher scores indicated more severe depressive symptoms. Because the response wording was adapted for the survey, the score was analyzed as a continuous symptom measure rather than using a diagnostic cutoff. Cronbach's alpha was 0.749 in the present sample.

#### 2.2.4 Bullying victimization

Students reported six forms of bullying victimization during the previous year: physical, verbal, relational, property-related, sexual, and cyber victimization. The six-domain framework was informed by the Chinese Delaware Bullying Victimization Scale-Student and BullyHARM-China (19, 20), although the present survey used one representative item per domain rather than either full instrument. Items were recoded to 0–3 and summed to create a bullying-severity score (range, 0–18; Cronbach's alpha = 0.764). A binary indicator of any bullying victimization was also created and defined as reporting at least one victimization type at a frequency of ‘occasionally' or more often.

#### 2.2.5 Suicidality

Suicidality was derived from three survey items assessing suicidal ideation, suicide preparation, and suicide attempt. Each item was rated from 1 (never) to 4 (frequently). Participants who endorsed any item at ‘occasionally' or more often were coded as having suicidality; those reporting ‘never' to all three items were coded as not having suicidality. Because the study materials did not identify these items as a standalone standardized suicide instrument, this composite was treated as an epidemiological indicator of suicide-related risk rather than a clinical diagnosis.

### 2.3 Covariates

Models adjusted for sex, grade, only-child status, ethnicity, residence, and subjective socioeconomic status (SES). Subjective SES was assessed using a 0–10 social-ladder item, with higher scores indicating a higher perceived family socioeconomic position, consistent with established adolescent subjective social-status approaches (21). Age was included in descriptive analyses but was not entered simultaneously with grade because of their substantial overlap.

### 2.4 Statistical analysis

Continuous variables are presented as means and standard deviations and categorical variables as counts and percentages. Demographic characteristics were compared across the four perceptual profiles using one-way analysis of variance or chi-square tests, as appropriate. Pearson correlations summarized bivariate associations between student-reported positive co-experiences and continuous mental-health outcomes. Convergence between homeroom-teacher ratings and class-mean student ratings was examined at the classroom level.

Because students were clustered within classes and shared the same homeroom-teacher rating, primary association analyses used generalized estimating equations (GEE) with class as the clustering unit and an exchangeable working correlation structure (22). Robust (sandwich) standard errors were used for all models. All 40 classes were included in each analysis, and outcome-specific complete-case analyses were used; analytic sample sizes are reported in the Results section for each outcome model. Gaussian distributions with identity links were specified for depressive symptoms and bullying severity, whereas binomial distributions with logit links were specified for any bullying victimization and suicidality. All models adjusted for sex, grade, only-child status, ethnicity, residence, and subjective SES. Results are reported as unstandardized beta coefficients or odds ratios (ORs) with 95% confidence intervals (CIs).

As a sensitivity analysis, teacher and student positive co-experience scores were standardized and a directional discrepancy score was calculated as delta = Z_teacher – Z_student. Participants in the highest quartile of delta were categorized as teacher markedly higher than student, and those in the lowest quartile as student markedly higher than teacher. The middle 50% were divided into concordant-high and concordant-low groups according to the mean standardized level of the two ratings, after which the GEE models were repeated. Outcome-specific complete-case analyses were used. All tests were two-sided, with *P* < 0.05 considered statistically significant. Analyses were conducted using Python 3.13 (Python Software Foundation; https://www.python.org/) and statsmodels 0.14.6 (statsmodels Developers; https://www.statsmodels.org/).

## 3 Results

### 3.1 Sample characteristics

The final analytic sample included 1,836 students (mean age = 13.61 years, SD = 1.52); 979 (53.3%) were male, 1,154 (62.9%) were in junior high school, 638 (34.7%) were only children, and 118 (6.4%) belonged to an ethnic minority group. Most participants lived in urban areas (*n* = 1,650, 89.9%), with 147 (8.0%) living in urban-rural fringe areas and 39 (2.1%) in rural areas. Mean subjective SES was 6.27 (SD = 1.50). The four perceptual profiles comprised 494 (26.9%) students in the both-high group, 435 (23.7%) in the teacher high-student low group, 390 (21.2%) in the both-low group, and 517 (28.2%) in the teacher low-student high group. The profiles differed significantly in age, grade distribution, and subjective SES, whereas sex, only-child status, ethnicity, and residence did not differ significantly (Table 1).

**Table 1:** Participant characteristics by teacher-student perceptual profile.

Characteristic	Total (*n* = 1,836)	Both high (*n* = 494)	Teacher high- student low (*n* = 435)	Both low (*n* = 390)	Teacher low- student high (*n* = 517)	*P* value
Age, years, mean (SD)	13.61 (1.52)	14.04 (1.42)	14.14 (1.31)	13.11 (1.50)	13.12 (1.54)	< 0.001
Female, *n* (%)	857 (46.7)	220 (44.5)	223 (51.3)	180 (46.2)	234 (45.3)	0.168
Junior high school, *n* (%)	1,154 (62.9)	249 (50.4)	201 (46.2)	305 (78.2)	399 (77.2)	< 0.001
Only child, *n* (%)	638 (34.7)	186 (37.7)	146 (33.6)	135 (34.6)	171 (33.1)	0.432
Ethnic minority, *n* (%)	118 (6.4)	33 (6.7)	23 (5.3)	27 (6.9)	35 (6.8)	0.740
Residence, *n* (%)						0.086
Urban	1,650 (89.9)	451 (91.3)	391 (89.9)	336 (86.2)	472 (91.3)	
Urban-rural fringe	147 (8.0)	34 (6.9)	38 (8.7)	43 (11.0)	32 (6.2)	
Rural	39 (2.1)	9 (1.8)	6 (1.4)	11 (2.8)	13 (2.5)	
Subjective SES, mean (SD)	6.27 (1.50)	6.26 (1.56)	6.07 (1.48)	6.23 (1.39)	6.47 (1.52)	0.001

The *P* value for residence refers to the overall chi-square test across the three residence categories. SES, socioeconomic status.

### 3.2 Descriptive statistics and correlations

Across the full sample, the mean student-reported positive co-experience score was 3.29 (SD = 0.69). Mean depressive-symptom and bullying-severity scores were 1.29 (SD = 1.44) and 1.52 (SD = 2.23), respectively. Among participants with complete outcome data, 942 of 1,809 (52.1%) reported at least one form of bullying victimization and 623 of 1,814 (34.3%) met the study definition of suicidality. The analytic sample sizes for the GEE models were *n* = 1,828 for depressive symptoms (8 missing), *n* = 1,818 for bullying severity (18 missing), *n* = 1,809 for any bullying victimization (27 missing), and *n* = 1,814 for suicidality (22 missing). At the classroom level, homeroom-teacher ratings showed little evidence of convergence with class-mean student ratings of positive co-experiences (*r* = −0.186, *P* = 0.250).

At the student level, positive co-experiences were negatively correlated with depressive symptoms (*r* = −0.223), bullying severity (*r* = −0.214), and suicide-related symptom severity (*r* = −0.155; all *P* < 0.001). The adverse mental-health outcomes were positively intercorrelated, with the strongest association observed between depressive symptoms and suicide-related symptom severity (*r* = 0.404, *P* < 0.001; Table 2).

**Table 2:** Pearson correlations between student-reported positive co-experiences and mental health outcomes.

Variable	Mean (SD)	1	2	3	4
1. Student-reported positive co-experiences	3.29 (0.69)	1			
2. Depressive symptoms	1.29 (1.44)	−0.223^***^	1		
3. Bullying severity	1.52 (2.23)	−0.214^***^	0.313^***^	1	
4. Suicide-related symptom severity	0.61 (1.17)	−0.155^***^	0.404^***^	0.391^***^	1

Suicide-related symptom severity was used only for the correlation analysis and was calculated by recoding the three suicide-related items to 0–3 and summing them. The primary regression outcome was binary suicidality.

^***^P < 0.001.

### 3.3 Perceptual profiles and mental health outcomes

The four perceptual profiles showed distinct unadjusted mental-health patterns. Mean depressive-symptom scores in the both-high, teacher high-student low, both-low, and teacher low-student high groups were 1.11, 1.74, 1.50, and 0.92, respectively. The corresponding prevalences of any bullying victimization were 48.9%, 55.1%, 61.6%, and 45.4%, and the prevalences of suicidality were 29.6%, 42.1%, 42.7%, and 26.2%. Thus, depressive symptoms were highest in the teacher high-student low group, bullying victimization was most pronounced in the both-low group, and the teacher low-student high group showed comparatively favorable outcomes.

These patterns were largely preserved after adjustment for sex, grade, only-child status, ethnicity, residence, and subjective SES (Table 3). Relative to the both-high group, the teacher high-student low group had higher depressive symptoms (beta = 0.580, 95% CI 0.395–0.765, *P* < 0.001), greater bullying severity (beta = 0.388, 95% CI 0.202–0.575, *P* < 0.001), higher odds of any bullying victimization (OR = 1.343, 95% CI 1.129–1.598, *P* < 0.001), and higher odds of suicidality (OR = 1.729, 95% CI 1.183–2.526, *P* = 0.005). The both-low group also had higher depressive symptoms (beta = 0.462, 95% CI 0.304–0.619, *P* < 0.001), greater bullying severity (beta = 0.570, 95% CI 0.160–0.980, *P* = 0.006), and higher odds of suicidality (OR = 1.782, 95% CI 1.240–2.560, *P* = 0.002), although its association with any bullying victimization did not reach statistical significance. The teacher low-student high group did not show elevated depressive symptoms or suicidality; its bullying-severity score was slightly lower than that of the both-high group (beta = −0.342, 95% CI −0.682 to −0.001, *P* = 0.049).

**Table 3:** Adjusted GEE associations between teacher-student perceptual profiles and adolescent mental health outcomes.

Perceptual profile	Depressive symptoms β (95% CI)	Bullying severity β (95% CI)	Any bullying victimization OR (95% CI)	Suicidality OR (95% CI)
Both high	Reference	Reference	Reference	Reference
Teacher high-student low	0.580 (0.395–0.765)^***^	0.388 (0.202–0.575)^***^	1.343 (1.129–1.598)^***^	1.729 (1.183–2.526)^**^
Both low	0.462 (0.304–0.619)^***^	0.570 (0.160–0.980)^**^	1.421 (0.873–2.314)	1.782 (1.240–2.560)^**^
Teacher low-student high	−0.085 (−0.272–0.102)	−0.342 (-0.682 to −0.001)^*^	0.749 (0.489–1.148)	0.850 (0.596–1.211)

Models adjusted for sex, grade, only-child status, ethnicity, residence, and subjective SES, with class specified as the clustering unit.

^*^P < 0.05.

^**^P < 0.01.

^***^P < 0.001.

The prespecified directional contrast showed that the two discordant profiles were not equivalent. Compared with the teacher low-student high group, students in the teacher high-student low group had depressive-symptom scores 0.665 points higher (95% CI 0.440–0.890, *P* < 0.001) and bullying-severity scores 0.730 points higher (95% CI 0.389–1.071, *P* < 0.001). Their odds of any bullying victimization and suicidality were 1.793 (95% CI 1.196–2.688, *P* = 0.005) and 2.035 (95% CI 1.364–3.036, *P* < 0.001), respectively, relative to the reverse-mismatch group.

### 3.4 Sensitivity analysis

Using the standardized directional-discrepancy definition, 459 students were classified as teacher markedly higher than student, 471 as student markedly higher than teacher, 453 as concordant high, and 453 as concordant low. The teacher-markedly-higher group had an unadjusted mean depressive-symptom score of 1.74, a bullying-victimization prevalence of 57.2%, and a suicidality prevalence of 43.5%; the corresponding values in the student-markedly-higher group were 0.91, 44.9%, and 26.0%.

After adjustment for the same covariates, the teacher-markedly-higher group continued to show poorer outcomes than the student-markedly-higher group: depressive symptoms were higher by 0.719 points (95% CI 0.478–0.960, *P* < 0.001), bullying severity was higher by 1.096 points (95% CI 0.742–1.450, *P* < 0.001), the odds of any bullying victimization were 2.408 times higher (95% CI 1.570–3.693, *P* < 0.001), and the odds of suicidality were 2.747 times higher (95% CI 1.766–4.273, *P* < 0.001; Table 4). The consistency of this directional pattern with the primary profile analysis reduced the likelihood that the findings were solely an artifact of the median cut points.

**Table 4:** Directional mismatch contrasts in the primary and sensitivity analyses.

Comparison	Depressive symptoms β (95% CI)	Bullying severity β (95% CI)	Any bullying victimization OR (95% CI)	Suicidality OR (95% CI)
Prespecified four-profile contrast: teacher high-student low vs. teacher low-student high	0.665 (0.440–0.890)^***^	0.730 (0.389–1.071)^***^	1.793 (1.196–2.688)^**^	2.035 (1.364–3.036)^***^
Extreme mismatch: teacher markedly higher vs. student markedly higher	0.719 (0.478–0.960)^***^	1.096 (0.742–1.450)^***^	2.408 (1.570–3.693)^***^	2.747 (1.766–4.273)^***^

Both contrasts were estimated using covariate-adjusted GEE models; covariates were identical to those in Table 3.

^**^P < 0.01.

^***^P < 0.001.

## 4 Discussion

Using paired reports from 1,836 adolescents and 40 homeroom teachers, this study examined whether teacher-student alignment in positive co-experiences was associated with adolescent mental health. Three findings stand out. First, homeroom-teacher ratings showed little evidence of convergence with class-mean student ratings. Second, relative to the both-high profile, both the teacher high-student low and both-low profiles were associated with poorer outcomes. Third, and most importantly, mismatch was strongly directional: compared with the teacher low-student high profile, the teacher high-student low profile was associated with greater depressive symptoms and bullying severity, 79% higher odds of bullying victimization, and approximately twice the odds of suicidality. The extreme-discrepancy sensitivity analysis reproduced and strengthened this pattern.

The directional finding is particularly informative when viewed through SDT/BPNT. SDT locates the psychological effects of social environments in the satisfaction or frustration of autonomy, competence, and relatedness (8, 9). Positive teacher-student co-experiences plausibly provide opportunities for relatedness through shared participation and emotional connection, and for competence through recognition of achievement and joint progress. However, these opportunities are not equivalent to experienced need support. In the present study, favorable homeroom-teacher appraisals were not associated with favorable mental-health outcomes when students themselves reported relatively few positive co-experiences. As a *post hoc* theoretical interpretation, this pattern is consistent with what may be termed a perceptual-translation proposition: support offered or perceived by the provider must be registered as supportive by the recipient before it can plausibly contribute to need satisfaction. This proposition extends the usual SDT sequence from social context to need-based experience by highlighting teacher-student perceptual alignment as a potentially important intermediate condition. Because psychological-need satisfaction was not measured and the design is cross-sectional, this interpretation should be considered theory-consistent rather than a direct test of mediation or causality.

The findings also provide a criterion-linked test of the OTM. Under the OTM, informant discrepancies become substantively informative when they show systematic relations with external criteria, rather than random divergence across reporters (14). Here, disagreement was not associated with uniformly elevated risk: the teacher high-student low profile consistently differed from the reverse mismatch across depression, bullying, and suicidality, and the same asymmetry emerged under the extreme-discrepancy definition. This pattern is compatible with the OTM concept of diverging operations and supports the broader argument that discrepancies in youth assessment should sometimes be preserved rather than averaged away (15). It is important to reiterate that the present discrepancy is cross-level rather than fully dyadic: homeroom teachers rated their overall interactions with the class as a whole, whereas students rated their own individual experiences, and all students within a class shared the same teacher-level rating. The results therefore indicate meaningful divergence between a class-level teacher appraisal and individual student experience, not that either reporter is objectively more accurate, and do not establish a one-to-one teacher-student perceptual mismatch.

The teacher high-student low profile may represent a particularly consequential support-to-experience gap. Ding et al. proposed that positive co-experiences strengthen teacher-student relationships through emotional bonding (12). A teacher may therefore perceive frequent shared learning, celebration, and progress at the class level while some students do not experience those events as personally inclusive, emotionally connecting, or competence affirming. The reverse profile is informative in this regard: students in the teacher low-student high group did not show elevated depression or suicidality and had comparatively low bullying severity. This asymmetry is consistent with the broader evidence that teacher-student relationship quality is strongly associated with student wellbeing and school functioning (5), while suggesting that the student's experienced relational environment may be more proximal to internalizing and safety-related outcomes than a teacher's global appraisal of the class.

The pattern across depression and suicidality also fits the broader SDT literature on need-based vulnerability, although the mechanism cannot be established in the present data. Teacher autonomy support has been linked prospectively to lower adolescent anxiety and depression through psychological-need satisfaction and school engagement (10), and need frustration has been associated with suicidal ideation through anxious-depressive symptoms in adolescents (11). In parallel, school connectedness is prospectively associated with lower depression and anxiety (6) and is generally protective in relation to suicidal ideation (7). The bullying findings further place perceptual mismatch within a wider school risk system: the both-low profile showed the highest unadjusted bullying prevalence, whereas the teacher high-student low profile remained at elevated risk despite a positive teacher appraisal. This is relevant because bullying victimization is a prominent environmental correlate of adolescent self-harm and suicidality (4), and Zhang et al.'s (13) entropy-based risk-network study from the same research program identified depression and bullying victimization as salient components of adolescent self-injury risk while also including positive teacher-student co-experiences as a social-context variable. Our findings add a multi-informant layer to that work by showing that the mental-health meaning of a positive relational context may depend on whether it is also experienced positively by students.

Alternative explanations are important. The cross-sectional design does not establish whether low student-perceived co-experiences precede mental-health difficulties or partly reflect them. Student-reported relational experiences and mental-health outcomes were assessed at the same time, and negative affect may shape attention to, interpretation of, or memory for interpersonal experiences. Supporting this possibility, a recent three-wave study by Zhang et al. (23) found that depressive symptoms prospectively predicted increased later recall of adverse childhood experiences. Although recalling childhood adversity differs from rating current school interactions, the study illustrates that affective state can shape reports of experience. Thus, the teacher high-student low profile should not be interpreted simply as teacher overestimation; it may reflect a combination of heterogeneous teacher-student experiences within classes, different reference frames, students' current emotional states, and genuine gaps in relational inclusion or support.

Future research should test the proposed integration of SDT and the OTM more directly. Longitudinal studies could obtain teacher ratings for specific students, repeated student ratings of positive co-experiences, and direct measures of autonomy, competence, and relatedness satisfaction and frustration. Such data would permit a multilevel pathway in which teacher-reported need-supportive behavior predicts student-perceived relational support, which in turn predicts need-based experiences and subsequent mental health. Methodologically, future studies should also retain the continuous joint distribution of teacher and student ratings. Polynomial regression with response surface analysis can distinguish level, congruence, and directional incongruence without relying on median splits. Zhang et al. recently used response surface analysis to characterize the combined and discrepant effects of childhood family abuse and peer bullying on adolescent mind-wandering (24); applying a similar framework to paired teacher-student ratings would provide a more stringent test of whether the present directional mismatch is linear, threshold-based, or concentrated at the extremes.

The findings also have practical implications for school mental-health assessment. A homeroom teacher's overall impression that teacher-student interactions are positive should not be assumed to represent every student's experience. Brief, confidential student feedback on relational experiences may usefully complement teacher observations, particularly when a student reports few positive co-experiences despite a favorable class-level teacher appraisal. Such discrepancies should be understood as a supplementary contextual indicator within a multi-source assessment framework, not as a standalone diagnostic tool or a basis for teacher accountability evaluation. They should not be used to label teachers or diagnose students. Rather, they may identify situations in which further assessment of depressive symptoms, peer victimization, perceived safety, and help-seeking opportunities is warranted. The practical implication is therefore not to privilege one informant, but to use the discrepancy itself as potentially informative data.

Taken together, the results connect two frameworks that are often considered separately. SDT explains why experienced social support may matter for adolescent adjustment, whereas the OTM explains why differences between reporters may themselves carry information. The present findings suggest a testable synthesis: the effectiveness of a potentially need-supportive school context may depend partly on successful perceptual translation from the provider's behavior or appraisal to the recipient's lived experience, and failures in that translation may be detectable through directional multi-informant mismatch.

### 4.1 Strengths and limitations

This study has several strengths. It paired students with their actual homeroom teachers and used parallel items to assess the same domain of positive co-experiences from two perspectives. It examined three clinically and educationally relevant outcomes–depressive symptoms, bullying victimization, and suicidality–and found a consistent direction of mismatch across them. The analyses accounted for classroom clustering using GEE, adjusted for multiple demographic covariates, and reproduced the main directional pattern using an alternative extreme-discrepancy definition.

Several limitations should be considered. First, the cross-sectional design precludes conclusions about temporal ordering or causality; all reported associations should be interpreted as cross-sectional correlations rather than causal effects. Second, student-reported co-experiences and mental-health outcomes share a common reporter, which may inflate some associations. Third, homeroom teachers rated their overall interactions with the class rather than their relationship with each individual student; the study therefore captured a cross-level perceptual mismatch between a class-level teacher appraisal and individual student experience, rather than a fully symmetric teacher-student dyadic discrepancy. Future studies should obtain teacher ratings for specific individual students. Fourth, the primary profile analysis dichotomized continuous scores at the median. Although this approach facilitated interpretation of directional profiles and the extreme-discrepancy sensitivity analysis yielded consistent results, dichotomization inevitably sacrifices information and may introduce boundary effects near the cutoff; continuous congruence methods such as polynomial regression with response surface analysis should be prioritized in future confirmatory work (24). Fifth, the teacher-level sample comprised only 40 classrooms and all participants were drawn from Hunan Province, with most students living in urban areas, which may limit generalizability. Finally, depressive symptoms and suicidality were assessed using brief survey-derived screening measures rather than structured clinical assessments; these outcomes should be interpreted as epidemiological risk indicators rather than clinical diagnoses, and the prevalence estimates should not be equated with clinically diagnosed rates.

## 5 Conclusion

Homeroom teachers and students did not necessarily share the same perception of positive teacher-student co-experiences, and the direction of this cross-level mismatch was associated with adolescent mental health in this cross-sectional sample. Adolescents whose homeroom teachers reported relatively high positive co-experiences while the students themselves reported relatively low levels showed greater depressive symptoms, bullying victimization, and suicidality than adolescents showing the reverse mismatch. These findings are jointly consistent with SDT's emphasis on experienced need support and the OTM view that informant discrepancies can contain meaningful contextual information. They further suggest a testable perceptual-translation hypothesis: relational support may be most protective when positive interactions are not only present or intended, but are also experienced as such by students. Perceptual mismatch may serve as a supplementary contextual indicator for school-based mental health screening, requiring multi-source information integration; it should not be used as a standalone clinical diagnostic tool or for teacher accountability evaluation. Integrating teacher and student perspectives may therefore reveal relational and mental-health risks that are not visible from class-level teacher impressions alone.

## Data Availability

The datasets presented in this study can be found in online repositories. The names of the repository/repositories and accession number(s) can be found at: https://www.kdocs.cn/l/caDmgILlcQ45.
